# Emergent Synchronization and Self-Organization of Autonomous Nanospinners

**DOI:** 10.1021/acs.nanolett.5c05293

**Published:** 2026-01-30

**Authors:** Tahniat Afsari, Suzanne Ahmed

**Affiliations:** Department of Nanoscience, Joint School of Nanoscience and Nanoengineering, University of North Carolina at Greensboro, 2907 East Gate City Boulevard, Greensboro, North Carolina 27401, United States

**Keywords:** Nanospinner, Chemical Propulsion, Synchronization, Self-Organization, Nanorobot

## Abstract

Autonomous, self-propelled, nanoscale particles, have sparked significant research interest due to their ability to serve as models for understanding the physical principles governing the individual and collective behaviors of living and nonliving systems at low Reynolds numbers. These systems also have many potential applications in drug delivery, environmental remediation, sensing, and oil recovery. However, most research has focused on particles undergoing linear motion, leaving a gap in the understanding of autonomous rotating systems. Addressing this gap would provide crucial insights into the nature of interactions and collective behaviors of rotating systems and enable applications such as nanomixing. In this work, we present the novel design and facile, high yield synthesis of autonomous nanospinners capable of achieving high frequency rotation. We report the evolution of their phase relationships, their emergent synchronization, and the self-organization of multiple spinners, marking a key step toward understanding the emergent behavior of autonomous nanoscale spinners.

## Graphical abstract

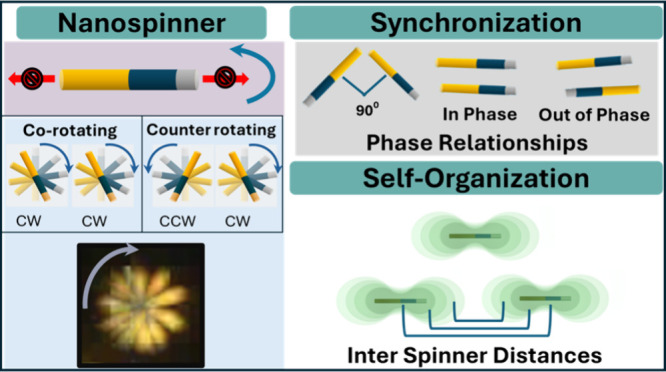

Self-propelled nanoscale particles, often called nanorobots, convert various forms of energy to mechanical motion.^1^ This motion can be powered by chemical fuel, or external fields such as magnetic, acoustic, electric, or electromagnetic fields.^2–15^ They can find utility in a range of applications including drug delivery, sensing, environmental remediation and oil recovery.^16–26^ They can be used as model systems for the understanding of the motion of biological entities on the same length scale.^27–29^ Among the most critical characteristics of biological systems is their ability to carry out collective behaviors that enable them to coordinate to evade predators or seek food.^30–32^ These collective behaviors emerge from independent autonomous entities mediated by, at the most basic level, nearest neighbor interactions.^33^ In recognition of this, collective behaviors have been explored with self-propelled particles.^34–38^ Yet, these studies have focused primarily on linearly moving entities.

Given the various applications that require rotational motion, including nanodrilling and mixing,^15,39–42^ effort has been directed at the design and synthesis of rotating nanorobots. Yet most of those synthesized thus far have been nonautonomous, relying on external forcing fields, most commonly magnetic fields, to move.^15,39,41–43^ Since all nonautonomous motors move in an identical manner, they cannot serve as model systems for the study of the emergence of collective behaviors in natural systems. For example, magnetic actuation is incapable of generating counter-rotating spinners to allow the evaluation of the evolution of the phase relationship between counter- versus co-rotating spinners.^43^ As such, to generate biomimetic systems, autonomous motion is required. Autonomous motion results from the local transduction of energy, such as the catalytic conversion of chemical fuel to motion at a particle surface, or the conversion of acoustic energy to motion due to particle shape asymmetry.^1,9^

While some autonomous nanoscale rotors have been synthesized,^8,9,44–46^ acoustically powered nanorotors exhibit little uniformity in their modes of motion with simultaneous helical, orbital and spinning modes.^8,9,44^ This eliminates the ability to explore the influence of rotational motion on emergent behaviors. Chemically powered nanorotors made so far have either spun too slowly to yield meaningful interactions, or had complex designs that required multistep, multi-instrument synthetic procedures that resulted in reduced yields, which hamper the study of emergent behaviors. Such studies would shed light on similar behaviors in natural systems, including for organisms that undergo rotational motion such as *Volvox*, or for organelles that individually rotate and collectively cooperate to generate motility including the flagella on *Escherichia coli* (*E. coli*) bacteria. Larger autonomous rotors based on chiral designs have been synthesized.^47–54^ However, in addition to their larger size; that may limit the range of applications they can be used for, they may not serve as meaningful models for smaller biological systems. Few studies on their interactions and collective behaviors have been reported.

Whereas theoretical and computational work has been done on the potential interactions and anticipated collective behaviors of rotational self-propelled particles,^55–64^ there remains a gap in the experimental work done to test these models. This is partly owing to the lack of a facile, high yield synthesis of chemically powered nanoscale rotors required for the systematic study of autonomous rotor interactions and collective behaviors.

In this work we take a key step toward addressing this gap. We present a simple achiral nanospinner design, with a facile synthesis for their fabrication. Their motion is powered by the catalytic breakdown of hydrogen peroxide fuel. In utilizing this prototype chemical reaction for the propulsion of self-propelled particles we have a system with a robust theoretical and computational framework for the understanding of our work and for future computational studies. We report their interactions, emergent phase synchronization and self-organization. We determine the relative phase evolution of spinners that are co-rotating versus those that are counter-rotating. We assess the dependence of interspinner distance on spinner rotational frequencies. The emergence of self-organization of multiple spinners to have consistent interspinner distances is quantified. Overall, this work reports on the emergent behavior and self-organization of autonomous nanoscale spinners.

Nanospinners are obtained via the synthesis of achiral trisegmented nanorods with the specific selection of the identity and order of the segments. Their design is based on trisegmented nanorod pullers where the outer two segments are anodes for the electrochemical decomposition of hydrogen peroxide, and the central segment serves as a cathode.^65,66^ In pullers, fluid flows inward from the edges of the rods and out at the center, nearly perpendicularly to the nanorod long axis.^65,66^ Nanorod-based pullers fabricated to date have been symmetric A–B–A nanorods where the two outer ends are made of the same material and hence are largely static beyond Brownian motion, and where motion is only induced upon assembly.^65,66^ This is due to the scallop theorem that governs motion at low Reynolds numbers and states that symmetry needs to be broken to yield motion.^67^ In this work, nanorod pullers are designed such that they are made of three different materials. By selecting three different materials, we produce a puller that is compositionally asymmetric, overcoming the scallop theorem enabling motion. The materials are ordered to produce two linear propulsion forces that are equal in magnitude but directed in opposite directions, suppressing translation; while producing an asymmetric flow around the rod, generating a torque on the rod and hence a consistent spinning motion. Spinning motion occurs when the center of mass of the rod remains largely still while it undergoes a rotational motion.( Figure 1). (See Supporting Information Videos S1, S2, and S3.)

**Figure 1:**
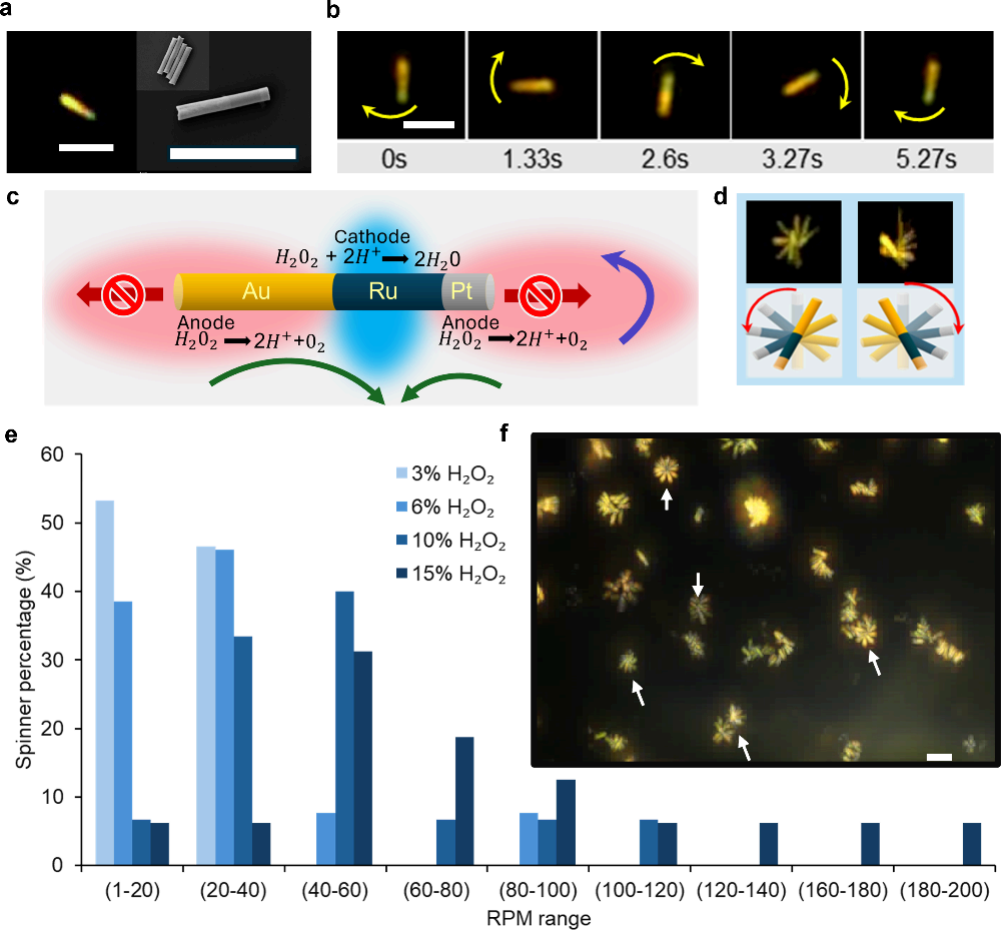
Nanospinner design and motion. (a, left) Optical microscope image of Au–Ru–Pt nanospinner showing the two different ends of the rod. Scale bar represents 5 μm. (a, right) Scanning electron microscopy micrograph of Au–Ru–Pt nanospinner. Inset: Multiple Au–Ru–Pt nanospinners. Scale bar represents 5 μm. (b) Image sequence showing the spinning motion of a nanospinner. Time stamps are indicated. Yellow arrows represent direction of rotation. Scale bar represents 5 μm. (c) Schematic of the Au–Ru–Pt nanospinner showing the reactions occurring at each segment. Red arrows represent linear motion that cancels out, green arrows represent the direction of fluid flow, and the purple arrow represents rotational motion. The red clouds on the Au and Pt anodes represent a positive charge due to proton production. The blue cloud on the Ru cathode represents a negative charge due to proton consumption. (d, top) Optical microscope images of bidirectional motion of an achiral nanospinner. Sequential frames are overlaid to show overall motion. (d, bottom) Schematics of overall motion and direction of motion. (e) Distribution of spinner speeds at different concentrations of hydrogen peroxide fuel. (f) Zoomed-out optical microscope image of rotating nanospinners. Sequential frames are overlaid to show overall motion. Some spinners indicated with white arrows. Scale bar represents 5 μm. See Supporting Information Videos S1, S2, and S3.

The spinners are gold–ruthenium–platinum (Au–Ru–Pt) nanorods. The outer Au and Pt segments act as anodes oxidizing hydrogen peroxide to produce oxygen gas and protons. The middle Ru segment acts as a cathode reducing hydrogen peroxide. The linear propulsion forces on opposite ends of the rod are balanced in magnitude and occur in opposite directions such that they cancel out. Bimetallic Au–Ru and Ru–Pt rods, the two opposing bimetallic segments of our trisegmented rod, have largely similar speeds at ∼22–26 and ∼26–30 μm s^–1^, respectively, where the propulsion force is directed, in both cases, toward the anode.^68^ Accounting for speed distributions within a single rod type, and the fact that for the Ru–Pt segment, anticipated to have a slightly faster linear bimetallic speed, the platinum segment is synthesized to have a smaller surface area, results in two ends with opposing speeds that they are essentially equivalent in magnitude. While linear motion is suppressed, fluid flows surrounding the rod are still active due to the surface chemical reactions. These flows are asymmetric on these asymmetric rods, generating a net torque resulting in the observed spinning rotational motion (Figure 1).

The trisegmented nanorods are synthesized via the electrodeposition of metal segments within porous anodic alumina templates within a single electrodeposition cell. Different metals are obtained via the sequential change of the deposition solution to one containing the desired metal ions. The diameter of the rods at 300 ± 15 nm is set by the diameter of the template pores, while the length is determined by the duration of the deposition. The Au–Ru–Pt rod segments are 1.5, 1, and 0.2 μm long, respectively (Figure 1). A single deposition yields approximately 2 × 10 ^9^ uniform rods.

These rods undergo a uniform spinning motion at all concentrations tested from 3% hydrogen peroxide to 15% hydrogen peroxide, and spin at speeds as high as 200 rpm. (See Supporting Information Video S1)

The torque on these nanospinners is calculated using eq 1 that relates the angular rotational frequency of the spinners (ω) and their torque (τ):^43^

$$\omega ∼\frac{\tau }{\pi \eta \sigma^{3}}$$ 1

where η is the viscosity of the fluid and σ is the rotation diameter of the spinners taken to be their length. For a rod rotating at 30 rpm, the value of the torque is approximately 1.7 × 10^–19^ J. This value is orders of magnitude larger than the thermal energy *k*_B_ *T*, which is approximately 4 × 10^–21^ J. Hence as expected, rotational motion is observed since the torque is larger than random thermal fluctuations. The drag force on the rods is evaluated by dividing the torque on the rods by the distance through which it acts, from the rod tip to its center. This yields a value of *F*_drag_ of approximately 0.13 pN.

The distribution of rotational frequencies at the various hydrogen peroxide concentrations tested is reminiscent of the Maxwell–Boltzmann distribution of speeds of an isotropic gas. Hence at the nanorod suspension densities studied in this work, the spinners are in the gaseous phase where fuel concentration and spinning speed is analogous to thermal excitation. This gas phase behavior is consistent with previous work on active particles at low suspension densities.^69,70^ Additionally, nanospinners show no preference for either clockwise or counterclockwise rotation. Interestingly, given the achiral nature of these spinners they are able to spontaneously reverse their direction of motion from clockwise to counterclockwise and vice versa (Figure 1, Video S2).

The basis of all emergent collective behaviors are nearest neighbor interactions. We interrogate the effect of spinning phase and rotational frequency on two-way nanospinner interaction behavior. Due to the asymmetry of the nanospinners, each one has a distinct phase. The phase angle of the spinner is defined as the angle the nanorod long axis makes with the *x*-axis, where the point of contact with the *x*-axis is the gold segment and where the angle is between 0 and 360°. The phase difference between two rods is the angle between them when their two gold segments are overlaid (Figure S1).

The evolution of the phase relationship between two interacting rods depends upon whether they are co-rotating or counter-rotating. Co-rotating rods demonstrate emergent in-phase synchronization, out of phase synchronization and a 90° phase difference: with each relationship lasting several cycles and occurring sequentially. In phase synchronization and out of phase synchronization occur when the difference in the phase angle between the two rods are 0 and 180°, respectively. The sequential phase transitions between different phase relationships occur suddenly over fractions of a cycle (Figure 2).

**Figure 2:**
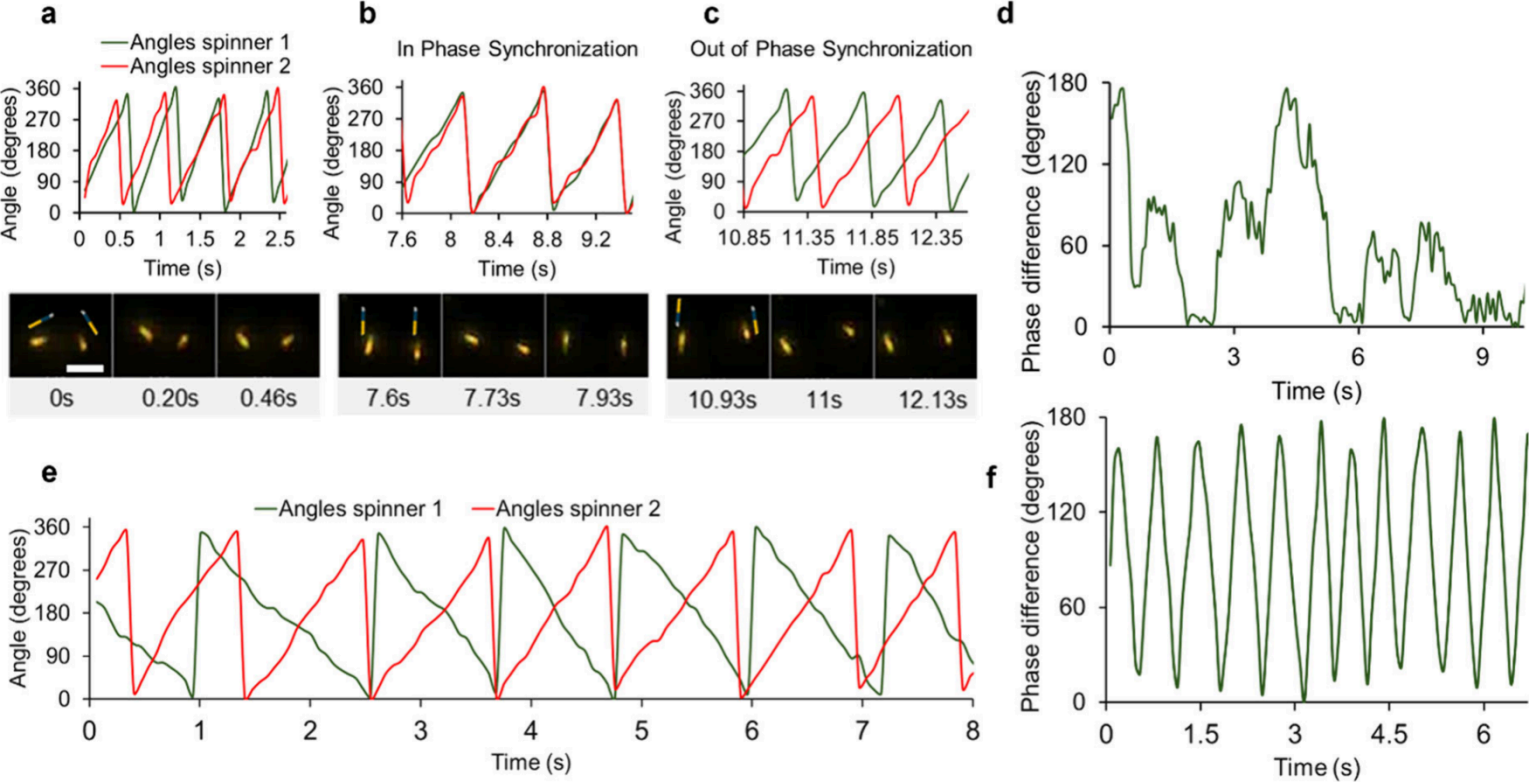
Evolution of nanospinner phase relationships for co-rotating and counter-rotating spinners. (a) 90° phase difference between a representative pair of co-rotating nanospinners. (Top) Phase angles of two co-rotating nanospinners. (Bottom) Optical image showing a 90° phase difference between co-rotating nanospinners with time stamps. Schematics of rods indicated. Scale bar represents 5 μm. (b) In phase synchronization of a representative pair of co-rotating nanospinners. (Top) Phase angles of co-rotating nanospinners showing a 0° phase difference. (Bottom) Optical image showing a 0° phase difference between co-rotating nanospinners with time stamps. Schematics of rods indicated. (c) Out of phase synchronization of a representative pair of co-rotating nanospinners. (Top) Phase angles of co-rotating nanospinners showing a 180° phase difference. (Bottom) Optical image showing a 180° phase difference between co-rotating nanospinners with time stamps. Schematics of rods indicated. (d) Evolution phase difference between a representative pair of co-rotating rods with time. (e) Phase difference between two counter-rotating nanospinners. (f) Evolution phase difference between counter-rotating rods with time. See Supporting Information Videos S4 and S5.

Counter-rotating rods on the other hand have a phase difference that oscillates regularly between an in phase and out of phase orientations sampling a continuous range of relative phase angles and where no phase locking is observed (Figure 2). Hence, rods spend approximately equal durations at different relative phase angle differences during a single cycle.

In terms of interspinner distances, upon approach at distances as far as four body lengths for rods spinning at 100 rpm, nanospinners fall within each other’s influence displaying a consistent average distance over the observation duration, sometimes it is as long as 50 full rotation cycles. This average interrod distance increases with increasing RPM (Figure 3). This observation is consistent with computational simulations on the interactions of rotating low Reynolds spinners. These simulations have shown that hydrodynamic repulsion increases with increasing rotational frequency, with the rotation of particles generating a fluid flow that repels neighboring particles.^43^ For autonomous nanoscale rotors, this is the first experimental verification of the increase in hydrodynamic repulsion of spinning particles with increased rotational frequency. Previous work on nanoscale and microscale spinners have utilized magnetic actuation, these are nonautonomous systems, and most importantly magnetic attraction between the particles upon approach cannot be eliminated, making it difficult to assess the influence of spinning frequency on interparticle interactions.^43^ Additionally, these studies, being on nonautonomous particles, have only considered co-rotating particles. Computational simulations on interacting rotors, focused on spherical particles, have shown that their fluid flow fields decay with 1/*r* ^2^,^43,57,58,64^ with *r* representing the distance from the rotor, influencing inter-rotor distances. In plotting the relationship between rotational frequency and the inverse squared of the average distance between the spinners, we find a robust fit, despite anticipated differences in the details of the flow fields of cylindrical rods (Figure 3a). The increase in interspinner separation with increased spinning frequency demonstrated in this work is indicative of the dominance of repulsive hydrodynamic forces on interactions, as spinners rotate more rapidly.

**Figure 3:**
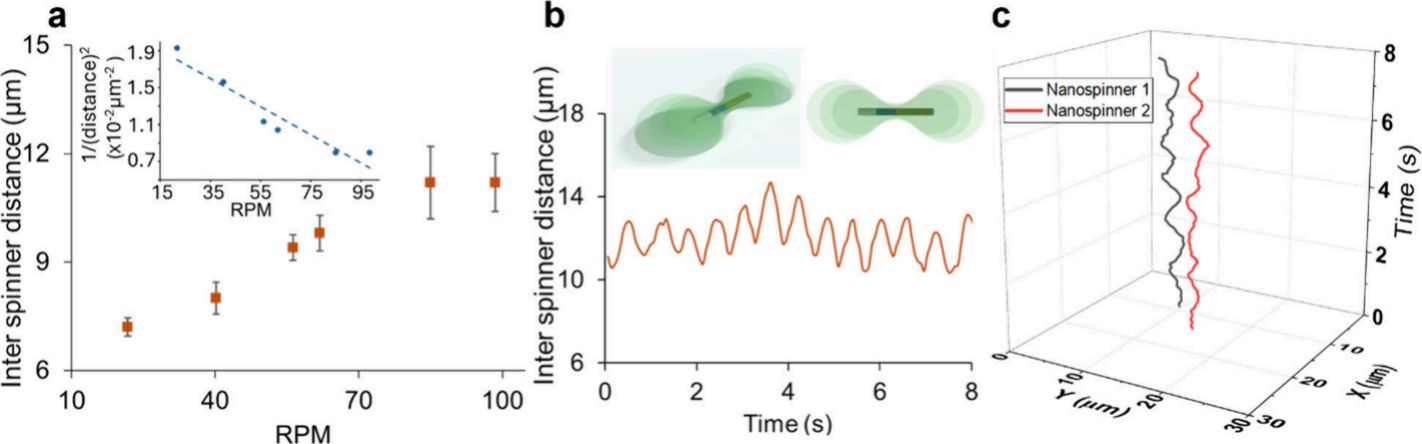
Inter-nanospinner distance. (a) Relationship between interspinner distance and the rotational frequency of nanospinners. Multiple spinner pairs were analyzed with an average of three to four pairs assessed per point, with the average RPM plotted, with over 21 pairs evaluated including both co- and counter-rotating rods. Inset: Plot of interspinner distance vs the inverse squared of the average distance between the rods. Plot demonstrates a linear fit with an *R* ^2^ value > 0.9. (b) Representative plot of interspinner distance of a single pair of nanospinners as a function of time. Insets: Schematic of the velocity of the fluid flow field surrounding the nanospinner where a darker green color indicates a faster speed. (c) Representative tracks of two interacting nanospinners as a function of time, demonstrating their oscillatory interparticle distance.

Additionally, individual pairs of interacting spinners display an oscillatory interspinner distance. This oscillation is regular, with a period correlated with the period of the rotation of the rods. Hence for rods spinning at 100 rpm, the cyclic increase and decrease in inter-rod distance occurs every 0.6 s (Figure 3). This oscillation in interparticle distance is also observed for a rapidly rotating spinner and a passive tracer particle (Video S6). This oscillation tends to be less regular due to the tracer particle’s higher Brownian motion in comparison to a second spinner, due to its lack of propulsion and smaller size (500 nm diameter).

This increase and decrease of interparticle distance can be attributed to the instantaneous shape of the velocity flow field generated by the rods. Computational studies have assessed that the velocity flow field for high aspect ratio particles has a dumbbell shape^71^ (Figure 3).

Intuitively the velocity of the fluid flowing adjacent to each portion of the rod is proportional to the tangential speed of the rod at that point. For a given rotational frequency, points furthest from the center of the rod move at higher tangential speeds than points closer to the center of the rod. This means that the fluid flow velocity at points closest to the ends of the rods tends to be higher and hydrodynamic repulsion consequently higher as well. Hence two spinners on approach, experience variable repulsion based on the region of the interacting flow field. When the rods interact at the flow fields at the two rod edges, maximum hydrodynamic repulsion and distance is observed, and when they interact at the flow fields near the center of the rods the shortest separations are observed. These increases and decreases occur with a period similar to that of the rotation period of the spinners, as is observed.

Self-organization with a consistent average distance between spinners emerges for multiple spinners (Figure 4). The average interspinner distance is calculated by determining the center of mass among the nanospinners at consecutive time points, done for each frame, followed by determining the distance from each spinner to the center of mass, and then taking the average of these distances, using eq 2.^72^

$$r\left(t\right)=\frac{1}{N}\overset{N}{\underset{i=1}{\sum }}\sqrt{({\left(x_{i}\left(t\right)-\mathrm{x̅}\right)}^{2}+({\left(y_{i}\left(t\right)-\mathrm{y̅}\right)}^{2}}$$ 2

Three, four, and five nanospinner clusters are stable over long durations of time and can typically last for the duration of the recording which is on the order of minutes.

**Figure 4:**
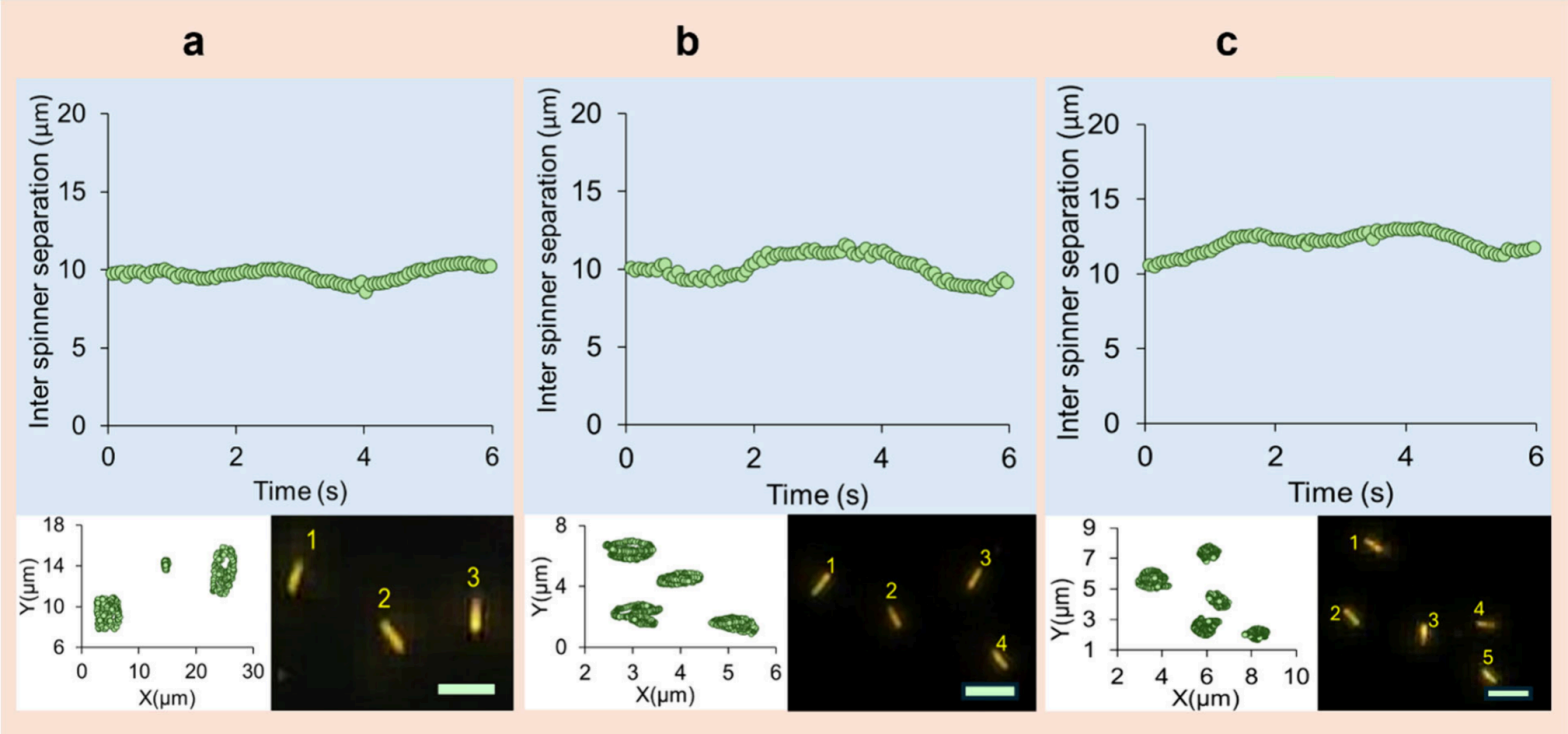
Emergent self-organization of nanospinners. (Top panels) Plots of the interspinner separation distance as a function of time. (Bottom left panels) Tracks of the nanospinners. (Bottom right panels) Optical images of nanospinners. Scale bars represent 5 μm. (a) Three nanospinners. (b) Four nanospinners. (c) Five nanospinners. See Supporting Information Videos S7, S8, and S9. The rotational speed of the nanospinners in panels a–c have an average value of ∼20 rpm resulting in similar average interspinner distances.

In this work, we report the high yield synthesis of autonomous, chemically actuated achiral nanoscale spinners. The spinners are trisegmented pullers of three different metallic materials electrodeposited in a single electrochemical cell. The spinners rotate at frequencies up to 200 rpm with a distribution of speeds reminiscent of the Maxwell–Boltzmann distribution of the speeds of a gas. We investigate their interactions, including the relative phase evolution of two spinners and the direct dependence of the interspinner distance on their rotational frequencies. We find that for co-rotating spinners phase synchronization emerges, while for counter-rotating spinners no extended phase locking occurs with the phase difference between spinners sampling all values. We observe that interspinner distances increase with an increase in spinning frequencies, confirming computational predictions and indicating the dominance of hydrodynamic repulsion with increasing speed. The emergence of self-organization of finite numbers of spinners to have consistent interspinner distances is quantified. Overall, we report on the emergent behaviors, including synchronization and self-organization, of autonomous nanoscale spinners.

## Supplementary Material





















## Data Availability

The data that support the findings of this study are available in the Supporting Information of this article.

## Notes

, videos of nanospinner motion and descriptions are available online. The Supporting Information is available free of charge at https://pubs.acs.org/doi/10.1021/acs.nanolett.5c05293.

Detailed methods (PDF)
Video S1 showing fast nanospinners (AVI)
Video S2 showing bidirectional rotation of spinner (AVI)
Video S3 showing zoomed-out view of multiple nanospinners (AVI)
Video S4 showing counter-rotating spinners (AVI)
Video S5 showing co-rotating spinners (AVI)
Video S6 showing spinner with tracer particle (AVI)
Video S7 showing three-multispinner self-organization (AVI)
Video S8 showing four-multispinner self-organization (AVI)
Video S9 showing five-multispinner self-organization (AVI)
