# Supplementary material for: Emergent Synchronization and Self-Organization of Autonomous Nanospinners
Source: Nano Lett. 2026 Jan 30;26(5):1682–8. doi: 10.1021/acs.nanolett.5c05293 (PMC12904068; doi:10.1021/acs.nanolett.5c05293)
Supplement: Supplementary file 1 [file nl5c05293_si_001.pdf]

Supporting Information for

Emergent Synchronization and Self-Organization of Autonomous Nanospinners

*Tahniat Afsari[1] and Suzanne Ahmed[1]**

[1]Department of Nanoscience, Joint School of Nanoscience and Nanoengineering, University of North Carolina at Greensboro, 2907 E Gate City Blvd, Greensboro, NC 27401

Video S1: Fast Nanospinners

Video shows AuRuPt nanospinners rotating at approximately 200RPM in 15% Hydrogen peroxide solution.

VideoS2: Bidirectional Rotation of Spinner

Video shows counterclockwise and clockwise bidirectional rotation of AuRuPt at 15% hydrogen peroxide solution.

Video S3: Zoomed out view of Multiple Nanospinners

Video shows an overview of many nanospinners in one frame rotating autonomously at different RPM at 15% hydrogen peroxide.

Video S4: Counter Rotating Spinners

Video shows interaction of two nanospinners rotating at different direction, one is clockwise, and another one is counterclockwise.

Video S5: Co-rotating Spinners

Video shows the interaction between two co-rotating nanospinners rotating in a same direction.

Video S6: Spinner with tracer particle

Video shows a single nanospinner interacting with a tracer particle (polystyrene beads, size 500 nm)

Video S7: Three Multi-spinner Self-Organization

Video shows 3 nanospinners at 15% hydrogen peroxide, where they maintain a constant average inter spinner separation during interaction indicating self-organization.

Video S8: Four Multi-spinner Self-Organization

The video shows 4 nanospinners at 15% hydrogen peroxide, where they maintain a constant average inter spinner separation during interaction indicating self-organization.

Video S9: Five Multi-spinner Self-Organization

Video shows 5 nanospinners at 15% hydrogen peroxide, where they maintain a constant average inter spinner separation during interaction indicating self-organization.

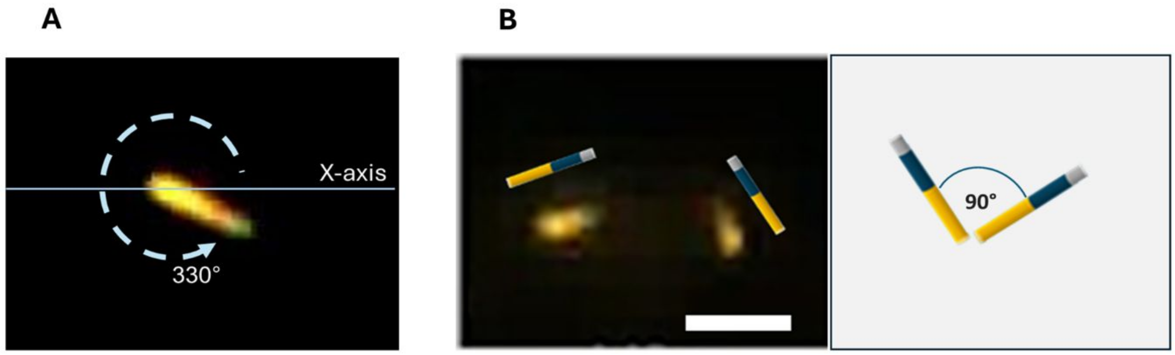

Determining Phase Angle and Phase Difference.

Figure S1.A. Determination of the phase angle of a single nanospinner. B. Determining the angle of the phase difference between two nanospinners.

Nanospinner angles with the x-axis were measured automatically utilizing the "Analyze Particles" feature in ImageJ. In the "Set Measurements" feature under the "Analyze" drop down menu "Fit ellipse" was selected. This fits an ellipse to the nanospinner in each frame and provides the angle it makes with the x-axis.

Detailed Methods:

Template Preparation. Nanorods were fabricated through sequential electrodeposition within the pores of commercial anodic alumina templates (Whatman Inc.), which have a nominal pore size of 0.2 µm, with the actual pore diameter being approximately 0.3 µm. To prepare the alumina templates for electrochemical deposition, a cathode layer was first evaporated onto one side of the template. The deposition process was carried out using a Kurt Lesker Physical Vapor Deposition (PVD) 75 system, which ensured precise control over the deposition of thin metal layers.

The process began with the deposition of a 5 nm titanium adhesion layer. Following this, a 350 nm thick silver layer was deposited on top of the titanium. The silver layer served as the cathode for the electrochemical deposition.

High-Yield Synthesis:

A platinum (Pt) wire was used as the anode. A sacrificial silver (Ag) layer was first electrodeposited, followed by sequential deposition of gold (Au), ruthenium (Ru), and platinum (Pt) segments. The deposition was carried out using commercial deposition solutions sourced from Technic, Inc. (Ag-1025 RTU, Au-Orotemp24 RTU, Ru, Pt- 2 g/L).

For the electrodeposition process, a constant deposition current was applied with specific time durations for each metal layer. The Ag was deposited at a constant cathodic current density of 2 mA/cm^2 for 25 minutes. Subsequently, Au and Ru were electrodeposited at a constant cathodic current of 1.4 mA/cm^2 for 16 minutes and 8 hours, respectively. The Pt segment was deposited at a constant cathodic current of 1 mA/cm^2 for 2.5 hours. This sequential deposition process enabled the controlled growth of the metal layers, resulting in the formation of well-defined nanostructures with distinct material compositions.

Nanorod Release:

The nanorods were released from the anodic alumina template and cathode through a selective etching process. Initially, the silver (Ag) cathode and sacrificial layer were removed by immersing the sample in a 1:1 nitric acid to water solution for 20 minutes, ensuring the dissolution of the metal layers. Following this, the alumina template was dissolved by soaking the sample overnight in a 2 M NaOH solution, which selectively etched the alumina, thereby liberating the nanorods from the template.

After the etching procedure, the nanorods were rinsed thoroughly with distilled water multiple times to remove any residual etchant. The resulting nanorods were then suspended in distilled water.

Nanorod Characterization:

For characterization, a small drop of the nanorod suspension was deposited onto a conductive silicon wafer. The nanorods were then imaged using a Field Emission Scanning Electron Microscope (FE-SEM, Zeiss Auriga FESEM).

Optical Observation and Tracking of Nanorod Motion:

Nanorods were placed within glass capillaries for imaging. Rectangular capillaries with dimensions of 300 μm × 3 mm cross-section and a total length of 50 mm were used. Capillaries with dimensions significantly larger than the dimensions of the rods were used and spinner viewing

was conducted away from capillary walls to eliminate the possibility or potential influence of edges. The capillary was initially filled with the nanorod suspension in different concentrations of hydrogen peroxide via capillary action. The concentrations of 3%, 6%, 10%, and 15% hydrogen peroxide were used.

The motion of the nanorods was observed using an Olympus BX53 microscope, equipped for optical imaging in dark field illumination. Imaging was performed with video capture at a frame rate ranging from 20 to 30 frames per second to ensure adequate temporal resolution of the nanorod motion. For tracking and analysis of the nanorod trajectories, the captured video frames were processed using ImageJ software. The software was employed to track individual nanorods, providing accurate measurements of their motion over time.
